# Recommendations of the Advisory Committee on Immunization Practices for Use of Cholera Vaccine

**DOI:** 10.15585/mmwr.mm6618a6

**Published:** 2017-05-12

**Authors:** Karen K. Wong, Erin Burdette, Barbara E. Mahon, Eric D. Mintz, Edward T. Ryan, Arthur L. Reingold

**Affiliations:** ^1^Division of Foodborne, Waterborne, and Environmental Diseases, National Center for Emerging and Zoonotic Infectious Diseases, CDC; ^2^Division of Infectious Diseases, Massachusetts General Hospital, Boston, Massachusetts; Harvard Medical School, Boston, Massachusetts; Department of Immunology and Infectious Disease, Harvard School of Public Health, Boston, Massachusetts; ^3^School of Public Health, University of California, Berkeley, California.

## Introduction

Cholera, caused by infection with toxigenic *Vibrio cholerae* bacteria of serogroup O1 (>99% of global cases) or O139, is characterized by watery diarrhea that can be severe and rapidly fatal without prompt rehydration. Cholera is endemic in approximately 60 countries and causes epidemics as well. Globally, cholera results in an estimated 2.9 million cases of disease and 95,000 deaths annually (*1*). Cholera is rare in the United States, and most U.S. cases occur among travelers to countries where cholera is endemic or epidemic. Forty-two U.S. cases were reported in 2011 after a cholera epidemic began in Haiti (*2*); however, <25 cases per year have been reported in the United States since 2012.

In 2016, lyophilized CVD 103-HgR (Vaxchora, PaxVax, Redwood City, California), a single-dose, live attenuated oral cholera vaccine, was approved by the Food and Drug Administration for the prevention of cholera caused by *V. cholerae* O1 in adults traveling to cholera-affected areas. Lyophilized CVD 103-HgR is the only cholera vaccine licensed for use in the United States. In June 2016, the Advisory Committee on Immunization Practices (ACIP) voted to recommend use of lyophilized CVD 103-HgR for prevention of cholera among adult travelers to areas with endemic or epidemic cholera caused by toxigenic *V. cholerae* O1, including areas with cholera activity during the last year that are prone to recurrence of cholera epidemics. ACIP considered evidence on safety and efficacy of the currently available formulation of CVD 103-HgR as well as that of a previously available formulation with identical phenotypic and genomic properties that was licensed and marketed in other industrialized countries before manufacture ceased in 2003 for business reasons (i.e., not because of safety or efficacy concerns) (*3*,*4*). This report provides new recommendations and guidance for vaccination providers and travelers about the use of lyophilized CVD 103-HgR. These recommendations apply to adults aged 18–64 years traveling to areas with endemic or epidemic cholera.

## Methods

ACIP work groups meet regularly to review all relevant data and prepare draft policy recommendations for ACIP consideration. Work groups are chaired by an ACIP member and include at least two ACIP members and a CDC subject matter expert; relevant ex officio members, liaison representatives, members of academia, other CDC staff members, and consultants are included as needed (*5*). In addition to ACIP members and CDC participants, the Cholera Vaccine Work Group (Work Group) includes participants from the Department of Defense, the Infectious Diseases Society of America, the National Foundation for Infectious Diseases, and academia. Members include experts in cholera, travel medicine, immunology, infectious diseases, obstetrics and gynecology, epidemiology, public health, military health, immunization safety, vaccine policy, and the Grading of Recommendations, Assessment, Development and Evaluation (GRADE) approach, a framework for evaluating scientific evidence. The Work Group convened monthly teleconferences starting in August 2015 to review cholera epidemiology and the evidence for the efficacy and safety of CVD 103-HgR according to the GRADE approach (https://www.cdc.gov/vaccines/acip/recs/grade/about-grade.html). During teleconferences, the Work Group reviewed and discussed a summary of findings and evidence quality for relevant outcomes. Questionnaires were used to collect and summarize Work Group opinions on key outcomes, evidence type, and proposed recommendations.

At the October 2015 ACIP meeting, the Work Group presented an overview of cholera epidemiology and CVD 103-HgR to ACIP. At the February 2016 meeting, the Work Group presented the GRADE review that summarized the strength of evidence for each of the outcomes assessed (prevention of cholera death, life-threatening cholera diarrhea, severe cholera diarrhea, and cholera diarrhea of any severity; induction of vibriocidal antibody response; occurrence of serious and systemic adverse events; and impact on effectiveness of co-administered vaccines and medications; (https://www.cdc.gov/vaccines/acip/recs/grade/cholera-CVD-103-HgR.html). At the June 2016 meeting, the Work Group presented proposed recommendations, and after a public comment period, ACIP voted to approve recommendations for use of lyophilized CVD 103-HgR. Postmarketing surveillance studies and additional data pertaining to use of the vaccine will be reviewed by ACIP as they become available, and recommendations will be updated as needed.

## Summary of Findings

Lyophilized CVD 103-HgR is the only cholera vaccine licensed for use in the United States. Its efficacy against severe diarrhea (defined here as fecal output >3 L/24 hours) after oral toxigenic *V. cholerae* O1 challenge is estimated to be 90% at 10 days after vaccination and 80% at 3 months after vaccination (*6*). Studies of the previously available formulation (discontinued in 2003) demonstrated similar efficacy (*7*). Both the previously and currently available formulations of the vaccine were effective in inducing a vibriocidal antibody response, the best available correlate of protection against cholera infection. No vaccine-related serious adverse events were reported in studies conducted using either of the two formulations. Studies with the currently available vaccine formulation found a slightly higher prevalence of diarrhea (mostly mild) among vaccine recipients (3.8%) than among unvaccinated groups (1.6%) (*8*). No other differences were detected between vaccinated and unvaccinated groups in the occurrence of any adverse events. Supporting evidence for the Work Group’s findings can be found online (*7*).

## Summary of Quality of Evidence Across Outcomes

The body of evidence, which included studies with the currently available lyophilized CVD 103-HgR formulation and studies with oral toxigenic *V. cholerae *O1 challenge, consistently indicated high vaccine efficacy and was judged to be GRADE evidence type 1 (evidence from randomized controlled trials or overwhelming evidence from observational studies), which is the strongest type of evidence. For safety outcomes, the data were more limited, because relatively few persons had received the currently available lyophilized vaccine formulation. Few studies evaluated coadministration of CVD 103-HgR with other vaccines or medications (*9*). Because of these limitations, the GRADE evidence for safety outcomes was judged to be type 3 (evidence from observational studies or randomized controlled trials with notable limitations).

## Summary of Rationale for Cholera Vaccine Recommendations

Assessment of the risk for cholera in U.S. travelers was addressed through review of the cholera epidemiology literature and expert judgment. Although cholera is rare among travelers returning to the United States from cholera-affected areas, and cholera is treatable if medical services are readily accessible, certain populations are at higher risk for toxigenic *V. cholerae *O1 infection and severe outcomes, and a traveler’s risk status is not always clear at the time of consultation.

### Risk for Exposure to Toxigenic *V. cholerae* O1

Persons at higher risk for exposure might include travelers visiting friends and relatives, health care personnel, cholera outbreak response workers, and persons traveling to or living in a cholera-affected area for extended periods (*10*–*13*). The primary prevention strategy for cholera is consistent access to and exclusive use of safe water and food and frequent handwashing. Nonetheless, travelers to areas of active cholera transmission, which include areas with current or recent endemic or epidemic cholera activity, might be exposed to toxigenic *V. cholerae* O1 through inadvertent or unexpected means, despite efforts to adhere to prevention measures.

### Risk for Poor Outcomes from Cholera

Cholera causes a profuse watery diarrhea leading to dehydration, which can be rapidly fatal unless reversed with fluid replacement therapy. Poor outcomes from toxigenic *V. cholerae* O1 infection might be more common in travelers with risk factors for severe disease, including the following: persons with blood type O; persons with low gastric acidity from antacid therapy, partial gastrectomy, or other causes; and travelers without ready access to medical services (*14*,*15*). Many travelers will not know their blood type at the time of consultation; however, an estimated 45% of persons in the United States have blood type O. Persons with medical conditions that would lead them to tolerate dehydration poorly, such as those with cardiovascular disease or kidney disease, might also be at increased risk for poor outcomes.

### Work Group Findings

Through the GRADE systematic review, the Work Group found high-quality evidence that the vaccine is highly effective and lower quality evidence that it is safe. The available safety data indicate no harms except for a slightly elevated risk for mild diarrhea among vaccine recipients. Although cholera is rare, the Work Group concluded that a safe and effective vaccine that can prevent a potentially severe cholera infection can benefit certain travelers.

## Recommendations for Prevention of Severe Cholera Among Travelers

### Personal Protective Measures

All travelers to cholera-affected areas should follow safe food and water precautions and proper sanitation and personal hygiene measures as primary strategies to prevent cholera. Travelers who develop severe diarrhea should seek prompt medical attention, particularly fluid replacement therapy.

### Use of CVD 103-HgR

CVD 103-HgR is recommended for adult travelers (aged 18–64 years) from the United States to an area of active cholera transmission. An area of active cholera transmission is defined as a province, state, or other administrative subdivision within a country with endemic or epidemic cholera caused by toxigenic *V. cholerae* O1 and includes areas with cholera activity within the last year that are prone to recurrence of cholera epidemics; it does not include areas where only rare imported or sporadic cases have been reported.

The vaccine is not routinely recommended for travelers who are not visiting areas of active cholera transmission. Most travelers from the United States do not visit areas with active cholera transmission (https://wwwnc.cdc.gov/travel/).

### Booster Doses

At this time, no data exist about the safety and efficacy of booster doses of lyophilized CVD 103-HgR for the prevention of cholera. The duration of protection conferred by the primary dose beyond the evaluated 3-month period is unknown. There is no recommendation for use of booster doses at this time.

### Coadministration of Other Medications or Vaccines

**Before cholera vaccination.** The Vaxchora package insert states that CVD 103-HgR should not be given to patients who have received oral or parenteral antibiotics in the preceding 14 days, because antibiotics might have activity against the vaccine strain. How long a person needs to be off antibiotics before receiving CVD 103-HgR is unknown; the duration will relate to the antimicrobial activity and half-life of the antimicrobial agent or agents. A duration of fewer than 14 days between stopping antibiotics and giving CVD 103-HgR might also be acceptable in certain clinical settings if travel is cannot be avoided before 14 days have elapsed after stopping antibiotics.

**During or after cholera vaccination.** A study of the previously available formulation of CVD 103-HgR found reduced immunogenicity when coadministered with chloroquine; thus, the manufacturer recommends that if chloroquine is indicated, it be started ≥10 days after CVD 103-HgR vaccination (*9*).

No data are available on concomitant administration of the currently available formulation of lyophilized CVD 103-HgR with other vaccines, including the enteric-coated oral live-attenuated typhoid vaccine (Ty21a, marketed as Vivotif). Based on expert opinion of how lyophilized CVD 103-HgR buffer might interfere with the enteric-coated Ty21a formulation, taking the first Ty21a dose ≥8 hours after ingestion of lyophilized CVD 103-HgR might decrease potential interference of the vaccine buffer with Ty21a vaccine.

The effect of oral or parenteral antibiotics given after vaccination with CVD 103-HgR is unknown; antibiotics might have activity against the vaccine strain and thus might reduce protection from vaccination. Most (83%) vaccine recipients have vibriocidal antibody seroconversion by 10 days after vaccination (*16*). Limited evidence suggests that some vaccine recipients who receive antibiotics ≤10 days after vaccination might still have vibriocidal antibody seroconversion (Lisa Danzig, PaxVax, personal communication, January 2017).

### Contraindications and Precautions for Use of Lyophilized CVD 103-HgR

**Allergy.** CVD 103-HgR should not be administered to persons with a history of severe allergic reaction, such as anaphylaxis, to any component of this vaccine or any cholera vaccine.

**Age.** No data currently exist about the safety and effectiveness of the currently available lyophilized CVD 103-HgR vaccine in children and teens aged <18 years or adults aged ≥65 years.

**Pregnancy and breastfeeding.** No data exist on use of CVD 103-HgR in pregnant or breastfeeding women. Pregnant women are at increased risk for poor outcomes from cholera infection. Pregnant women and their clinicians should consider the risks associated with traveling to areas of active cholera transmission. The vaccine is not absorbed systemically; thus, maternal exposure to the vaccine is not expected to result in exposure of the fetus or breastfed infant to the vaccine. However, the vaccine strain might be shed in stool for ≥7 days after vaccination, and theoretically, the vaccine strain could be transmitted to an infant during vaginal delivery.

**Immunocompromised persons.** No data exist on use of the currently available lyophilized CVD 103-HgR formulation in immunocompromised populations. A study of the previously available CVD 103-HgR formulation among HIV-positive adults in Mali found that vibriocidal seroconversion was slightly lower among HIV-positive than HIV-negative participants (58% versus 71%) (*17*). No significant differences in occurrence of any systemic adverse events were found between vaccinated and comparison populations.

**Shedding and transmission.** Lyophilized CVD 103-HgR is an oral live attenuated vaccine that can be shed in stool and potentially transmitted to close contacts. The vaccine strain was cultured from stool in 11.1% of vaccine recipients in the 7 days after vaccination with the previously available formulation (*16*). The currently available formulation of lyophilized CVD 103-HgR was not isolated from the stools of 28 household contacts whose stool was cultured 7 days after vaccination (*16*), and few (<1%) household contacts of persons vaccinated with the previously available CVD 103-HgR formulation had the vaccine strain isolated from stool cultured 5 days after vaccination. However, later transmission could have been missed. A study with the previously available vaccine formulation detected seroconversion among 3.7% of family contacts of vaccine recipients at 9 or 28 days after vaccination (*18*).

## Reporting of Vaccine Adverse Events and Additional Information

Because surveillance for rare adverse events will add to information about the safety of CVD 103-HgR, all clinically significant adverse events should be reported to the Vaccine Adverse Events Reporting System at https://vaers.hhs.gov or at 1-800-822-7967. To enroll in a registry monitoring pregnancy outcomes in women exposed to lyophilized CVD 103-HgR, contact PaxVax at 1-800-533-5899. Additional information about cholera and CVD 103-HgR is available at https://www.cdc.gov/cholera/index.html.

## References

[1] Ali M, Nelson AR, Lopez AL, Sack DA. Updated global burden of cholera in endemic countries. PLoS Negl Trop Dis 2015;9:e0003832. 10.1371/journal.pntd.0003832 26043000 PMC4455997

[2] CDC. Cholera and other *Vibrio* illness surveillance (COVIS). Atlanta, GA: US Department of Health and Human Services, CDC; 2016. https://www.cdc.gov/vibrio/surveillance.html

[3] Herzog C. Successful comeback of the single-dose live oral cholera vaccine CVD 103-HgR. Travel Med Infect Dis 2016;14:373–7. 10.1016/j.tmaid.2016.07.003 27425792

[4] Levine MM, Chen WH, Kaper JB, Lock M, Danzig L, Gurwith M. PaxVax CVD 103-HgR single-dose live oral cholera vaccine. Expert Rev Vaccines 2017;16:197–213. 10.1080/14760584.2017.1291348 28165831

[5] Smith JC. The structure, role, and procedures of the U.S. Advisory Committee on Immunization Practices (ACIP). Vaccine 2010;28(Suppl 1):A68–75. 10.1016/j.vaccine.2010.02.037 20413002

[6] Chen WH, Cohen MB, Kirkpatrick BD, Single-dose live attenuated oral cholera vaccine (CVD 103-HGR) protects against cholera at 10 days following vaccination: results of a *Vibrio cholerae* O1 El Tor Inaba challenge study. In: proceedings of the 63rd Annual Meeting of the American Society of Tropical Medicine and Hygiene, 2013. New Orleans, Louisiana. http://onlinelibrary.wiley.com/o/cochrane/clcentral/articles/363/CN-01056363/frame.html.

[7] CDC. Obtaining and evaluating evidence with grading of recommendations, assessment, development and evaluation (GRADE) for lyophilized CVD 103-HgR vaccine. Atlanta, GA: US Department of Health and Human Services, CDC; 2017. https://www.cdc.gov/vaccines/acip/recs/grade/cholera-CVD-103-HgR.html

[8] Advisory Committee on Immunization Practices. Summary Report, February 24, 2016. Atlanta, GA: US Department of Health and Human Services, CDC, Advisory Committee on Immunization Practices; 2016. https://www.cdc.gov/vaccines/acip/meetings/downloads/min-archive/min-2016-02.pdf, editor.2016

[9] Kollaritsch H, Furer E, Herzog C, Wiedermann G, Que JU, Cryz SJ Jr. Randomized, double-blind placebo-controlled trial to evaluate the safety and immunogenicity of combined *Salmonella *Typhi Ty21a and *Vibrio cholerae* CVD 103-HgR live oral vaccines. Infect Immun 1996;64:1454–7. 8606118 10.1128/iai.64.4.1454-1457.1996 PMC173943

[10] Loharikar A, Newton AE, Stroika S, Cholera in the United States, 2001–2011: a reflection of patterns of global epidemiology and travel. Epidemiol Infect 2015;143:695–703. 10.1017/S0950268814001186 24865664 PMC4615619

[11] Haus-Cheymol R, Theodose R, Quilici ML, A cluster of acute diarrhea suspected to be cholera in French travelers in Haiti, December 2010. J Travel Med 2012;19:189–91. 10.1111/j.1708-8305.2012.00607.x 22530828

[12] Schilling KA, Cartwright EJ, Stamper J, Diarrheal illness among US residents providing medical services in Haiti during the cholera epidemic, 2010 to 2011. J Travel Med 2014;21:55–7. 10.1111/jtm.12075 24383654

[13] Taylor DN, Rizzo J, Meza R, Perez J, Watts D. Cholera among Americans living in Peru. Clin Infect Dis 1996;22:1108–9. 10.1093/clinids/22.6.1108 8783724

[14] Glass RI, Holmgren J, Haley CE, Predisposition for cholera of individuals with O blood group. Possible evolutionary significance. Am J Epidemiol 1985;121:791–6. 10.1093/oxfordjournals.aje.a114050 4014172

[15] Bavishi C, Dupont HL. Systematic review: the use of proton pump inhibitors and increased susceptibility to enteric infection. Aliment Pharmacol Ther 2011;34:1269–81. 10.1111/j.1365-2036.2011.04874.x 21999643

[16] Chen WH, Greenberg RN, Pasetti MF, Safety and immunogenicity of single-dose live oral cholera vaccine strain CVD 103-HgR, prepared from new master and working cell banks. Clin Vaccine Immunol 2014;21:66–73. 10.1128/CVI.00601-13 24173028 PMC3910924

[17] Perry RT, Plowe CV, Koumaré B, A single dose of live oral cholera vaccine CVD 103-HgR is safe and immunogenic in HIV-infected and HIV-noninfected adults in Mali. Bull World Health Organ 1998;76:63–71. 9615498 PMC2305629

[18] Simanjuntak CH, O’Hanley P, Punjabi NH, Safety, immunogenicity, and transmissibility of single-dose live oral cholera vaccine strain CVD 103-HgR in 24- to 59-month-old Indonesian children. J Infect Dis 1993;168:1169–76 .PubMed 10.1093/infdis/168.5.1169 8228350

